# Aerobic fitness associates with mnemonic discrimination as a mediator of physical activity effects: evidence for memory flexibility in young adults

**DOI:** 10.1038/s41598-017-04850-y

**Published:** 2017-07-11

**Authors:** Kazuya Suwabe, Kazuki Hyodo, Kyeongho Byun, Genta Ochi, Takemune Fukuie, Takeshi Shimizu, Morimasa Kato, Michael A. Yassa, Hideaki Soya

**Affiliations:** 1 Laboratory of Exercise Biochemistry and Neuroendocrinology, Faculty of Health and Sport Sciences, University of Tsukuba, Ibaraki, 305-8574 Japan; 2 Department of Sports Neuroscience, Advanced Research Initiative for Human High Performance (ARIHHP), Faculty of Health and Sport Sciences, University of Tsukuba, Ibaraki, 305-8574 Japan; 3 Physical Fitness Research Institute, Meiji Yasuda Life Foundation of Health and Welfare, Tokyo, 192-0001 Japan; 4 Sports Research & Development Core, University of Tsukuba, Ibaraki, 305-8574 Japan; 5 Department of Health and Nutrition, Yonezawa Nutrition University of Yamagata Prefecture, Yonezawa, 992-0025 Japan; 6 Department of Neurobiology and Behavior, Center for the Neurobiology of Learning and Memory, University of California, Irvine, 92697-3800 CA USA

## Abstract

A physically active lifestyle has beneficial effects on hippocampal memory function. A potential mechanism for this effect is exercise-enhanced hippocampal plasticity, particularly in the dentate gyrus (DG). Within hippocampal memory formation, the DG plays a crucial role in pattern separation, which is the ability to discriminate among similar experiences. Computational models propose a theoretical hypothesis that enhanced DG-mediated pattern separation leads to “memory flexibility”–a selective improvement in the ability to overcome moderate levels of mnemonic interference. Thus, in the current cross-sectional study of healthy young adults, we tested the working hypothesis that aerobic fitness, as a physiological indicator of endurance capacity associated with physical activity, is strongly associated with mnemonic discrimination at moderate interference levels. When divided the sample (n = 75) based on a median split of aerobic fitness, the higher fitness group had better discrimination performance for moderate interference levels compared to the lower fitness group, namely, exhibited memory flexibility. Moreover, aerobic fitness levels were positively associated with discrimination performance for moderate interference levels, as a mediator of physical activity effects. This evidence suggests that aerobic fitness levels are associated with hippocampal DG-related memory, which is consistent with literature showing positive effect of physical exercise on hippocampal memory.

## Introduction

A physically active lifestyle has beneficial effects not only on physical fitness, but also on brain and cognitive health. Indeed, abundant evidence has demonstrated that aerobic fitness level, which is a physiological indicator of endurance capacity associated with physical activity, is positively associated with various aspects of cognitive functioning across all ages^1, 2^. Several recent studies have demonstrated that higher levels of aerobic fitness are associated with superior hippocampal memory performance and that this relationship is partially mediated by hippocampal volume in children and older adults^3, 4^.

A potential mechanism for this association is exercise-enhanced neural plasticity, particularly in the dentate gyrus (DG) of the hippocampus. A number of animal studies, including ours, have revealed that long-term exercise intervention has a positive impact on cell proliferation and survival in the DG and improves learning and memory^5–9^. One human study indirectly supports these findings: aerobic exercise training increased cerebral blood volume in the DG, which is related to neurogenesis, and this effect was positively correlated with aerobic fitness enhancement^10^. This evidence led us to directly test the hypothesis that aerobic fitness, as a physiological landmark of habitual physical activity, may be strongly associated with DG-related memory function.

It has been demonstrated that DG granule cells are sensitive to mnemonically similar experience and are able to generate different representations even with minimal changes in sensory input^11^. In computational models, this process is known as pattern separation; it plays a crucial role in overcoming mnemonic interference and is a fundamental component of episodic memory^12^. Pattern separation allows for similar experiences to be stored using distinct representations depending on the extent to which they generate interference. Interestingly, along with a reduction of DG-mediated pattern separation with aging, the relationship between mnemonic interference levels and discrimination performance becomes increasingly nonlinear, following an exponential curve (“memory rigidity”) and discrimination performance is more impaired at moderate interference levels (for review, see Leal and Yassa^13^). Conversely, computational models propose a counterpart theoretical concept of “memory flexibility”–that is, enhanced DG-mediated pattern separation leads to a curvilinear the natural logarithmic relationship between discrimination performance and an enhanced ability to overcome moderate levels of interference.

We hypothesized that if physical-activity-enhanced aerobic fitness is related to DG-mediated pattern separation, then aerobic fitness should mediate the relationship between physical activity and discrimination performance, and be selectively associated with discrimination performance for moderate level of interference, thereby leading to a more natural logarithmic curve input/output transformation. To test this hypothesis, we first examined differences in discrimination performance between higher and lower fitness young adults. Moreover, we examined the mediation effect of aerobic fitness on the association of physical activity levels with discrimination performance. Although, we assessed aerobic fitness and physical fitness in a cross-sectional manner, we focused on aerobic fitness, which is often considered an objective landmark of habitual physical activity^14^, as a mediator of the association between physical fitness and discrimination performance. To assess mnemonic discrimination performance, we adopted a mnemonic discrimination task which uses lures that vary in similarity, thereby parametrically manipulating interference^15, 16^. The lure discrimination score, which is the proportion of correct rejection of lure items, for the task is strongly tied to age-related changes in discrimination performance^15–17^ and functional signals in the DG/CA3^18^. Thus, the task and its corresponding lure discrimination measure are appropriate for assessing an individual capacity for DG-related memory function.

## Materials and Methods

### Participants

Seventy-five healthy young adults aged 18–24 years (mean age 20.2 ± 1.6 years, 30 females) participated in this study. All participants were recruited from student population of the university of Tsukuba using ethics board approved flyers that were posted across the campus. No subject reported a history of neurological or psychiatric disorders, or had a disease requiring medical care. All participants had normal or corrected-to-normal vision at least 20/40, and normal color vision. Participants’ demographic and physiological characteristics are presented in Table 1.

**Table 1:** Participant demographics, physiological characteristics and behavioral data.

Measure	All	High-Fit	Low-Fit	P-value
Sample Size	75 (30 female)	37 (15 female)	37 (15 female)	
Age[yr]	20.2 (1.57)	20.1 (1.51)	20.4 (1.62)	0.376
Height [cm]	165.5 (8.10)	165.0 (7.82)	165.9 (8.55)	0.633
Weight [kg]	59.3 (11.13)	56.9 (8.55)	61.4 (12.88)	0.079
BMI [kg/m^2^]	21.5 (2.93)	20.8 (1.82)	22.2 (3.57)	0.041
BDI-2	6.5 (4.75)	6.3 (5.17)	6.8 (4.36)	0.640
**IPAQ**				
TPA [METs-hour/wk]	29.7 (18.21)	36.1 (16.46)	22.4 (16.85)	<0.001
**Graded exercise test**				
$\dot{{\rm{V}}}$O_2peak_ [ml/kg/min]	41.7 (7.55)	46.4 (5.94)	36.9 (5.93)	<0.001
HR_peak_ [bpm]	176.8 (12.79)	175.3 (12.54)	177.8 (12.95)	0.427
WR_peak_ [Watt]	217.7 (51.80)	227.3 (46.44)	205.2 (52.93)	0.067
**Mnemonic Discrimination Task**				
Targets				
Old	0.71 (0.12)	0.71 (0.12)	0.71 (0.12)	0.882
Similar	0.15 (0.10)	0.16 (0.12)	0.13 (0.08)	0.221
New	0.13 (0.09)	0.12 (0.09)	0.15 (0.10)	0.223
Lures				
Old	0.37 (0.14)	0.33 (0.13)	0.40 (0.15)	0.067
Similar	0.40 (0.18)	0.44 (0.20)	0.36 (0.15)	0.052
New	0.22 (0.11)	0.21 (0.10)	0.23 (0.11)	0.359
Foils				
Old	0.04 (0.05)	0.04 (0.03)	0.05 (0.06)	0.363
Similar	0.09 (0.07)	0.09 (0.07)	0.09 (0.07)	0.958
New	0.86 (0.09)	0.86 (0.09)	0.85 (0.09)	0.652
Target Recognition	0.67 (0.12)	0.67 (0.13)	0.67 (0.11)	0.832
Discrimination Score				
High_Sim	0.49 (0.16)	0.51 (0.16)	0.46 (0.16)	0.126
Mid_Sim	0.65 (0.17)	0.69 (0.14)	0.60 (0.18)	0.018
Low _Sim	0.77 (0.14)	0.78 (0.13)	0.75 (0.16)	0.371

Note: BMI = Body Mass Index; BDI = Beck Depression Inventory; IPAQ = International Physical Activities Questionnaire; TPA = Total Physical Activity; METs = Metabolic Equivalent of Task; $\dot{{\rm{V}}}$O_2peak_ = Peak Oxygen uptake; HR = Heart Rate; WR = Work Rate. Values are mean (SD). Behavioral data indicate response proportion of each trial by each response type. P-values indicate the P-values for the independent t-tests comparing High-Fit vs. Low-Fit. $\dot{{\rm{V}}}$O_2peak_ range for male: Low-Fit (n = 22) 30.7–46.1, High-Fit (n = 22) 46.6–57.8; female: Low-Fit (n = 15) 27.3–35.6, High-Fit (n = 15) 36.9–45.1.

All experimental protocols were approved by the Institutional Ethics Committee of the University of Tsukuba. The experiment was carried out in accordance with those protocols and guidelines of the latest version of the Helsinki Declaration. Written informed consent was obtained from all participants.

### Experimental procedure

The participants visited our laboratory twice. On the first day, they completed health/demographic questionnaires, and performed a graded exercise test to assess their maximal aerobic fitness level. On the second day, at least 48 hours after the fitness assessment, all participants underwent a mnemonic discrimination task. On both experimental days, they were asked to refrain from exercise and the consumption of alcohol and caffeine for at least 24 hours prior to the experiment so as to control for outside factors that could affect cardiovascular and cognitive function.

### Physical activity assessment

The Japanese language version of the International Physical Activities Questionnaire (IPAQ)-long form was used as a measure of self-reported physical activity^19^. Participants were asked to report the amount of walking, and the number of times they performed moderate and vigorous activities over the previous seven days. Total physical activity (TPA) was calculated as metabolic equivalent of task (MET)-hours/week by summing weekly hours of each reported physical activity weighted by MET values^20^.

### Cardiorespiratory aerobic fitness assessment

Individual aerobic fitness level was assessed using a graded exercise test with a recumbent ergometer (Strength-ergo 240, Mitsubishi Electric Corporation, Japan). Peak oxygen uptake ($\dot{{\rm{V}}}$O_2peak_), the gold-standard measurement of aerobic fitness, was determined by measuring oxygen uptake continuously during an incremental test to exhaustion. After warming up for 3 minutes at 30 W, the work rate increased by 20 W (females: 15 W) per minute in a constant and continuous manner to exhaustion. The pedaling rate was kept at 60 rpm. Exhaled gas was analyzed using a gas analyzer (Aeromonitor AE280S, Minato Medical Science, Japan). Heart rate (HR) and rating of perceived exertion (RPE) were recorded every minute. The RPE was assessed verbally on which participant are asked to rate their perceived exertion ranging from 6 (no exertion at all) to 20 (maximal exertion). $\dot{{\rm{V}}}$O_2peak_ was determined when at least two of the following criteria were satisfied: (1) the respiratory exchange ratio (R) exceeded 1.05, (2) achievement of 90% of age-predicted peak HR (220–age), and (3) an RPE of 19 or 20.

### Mnemonic discrimination task

**Figure 1:**
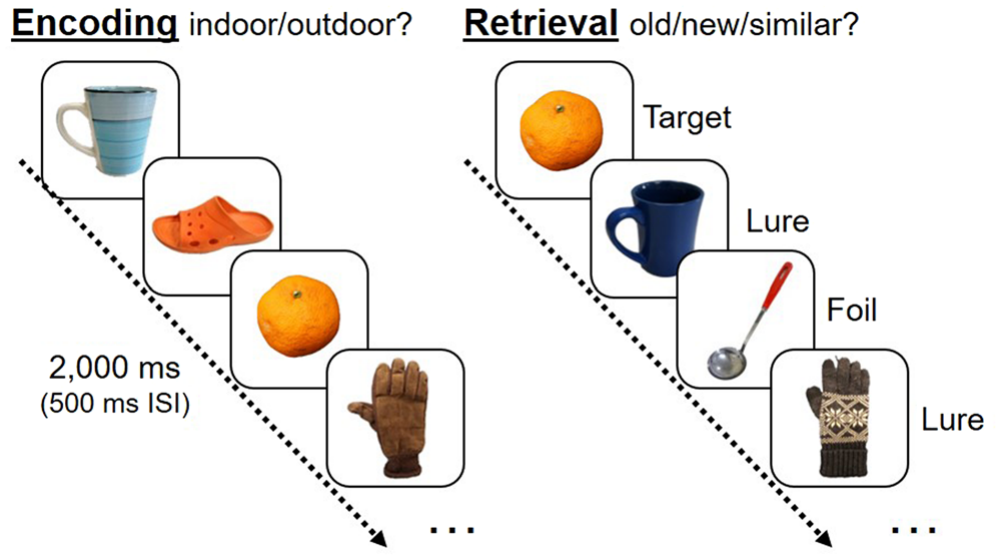
Mnemonic Discrimination Task. Encoding phase was an indoor/outdoor judgment task. Recognition was an old/similar/new judgment task using targets, foils and similar lures.

The task used in this study consisted of an encoding phase and a retrieval phase (Fig. 1). During the encoding phase, participants were shown a series of 196 color photographs of everyday objects on a white background on a computer screen. They were required to judge whether the presented picture was an indoor or outdoor object. After the encoding phase, the participants rested for 45 minutes while they watched a movie (low arousal stimulus) without sound to avoid sleeping. After rest, the participants underwent the retrieval phase, during which a series of 256 items were randomly displayed on a monitor and participants identified each item as “old”, “similar”, or “new” by pressing buttons. One-fourth of the stimuli in the retrieval phase were “old”, or exact repetitions of stimuli presented in the encoding phase (64 targets); half of the stimuli were “similar” to those seen during the encoding phase, but not identical (128 lures); and one-fourth of the stimuli were “new” stimuli not previously seen (64 foils). In both phases, each picture was presented for 2 s with an inter-stimulus interval (ISI) of 0.5 s. All participants underwent a practice session (4 encoding items; 8 retrieval items) to ascertain their understanding of task instructions and procedures.

This task measures discrimination performance as a function of interference levels. The lure stimuli were sorted into three bins based on the degree of mnemonic similarity to the targets, with high-, middle- and low-similarity lures^16^. To assess discrimination performance across all trial types and each similarity lure, we calculated the discrimination score (DS) as the inverse of the probability of responding with “old”, representing “similar” or “new” responses (1−p (“old”|Type)). We adopted this measure to factor out response bias of using “similar” or “new” for lure bins across participants. It should be noted that the DS for target trials represents the propensity for incorrect rejection since the correct response for this trial is “old”. In addition, we calculated target recognition score as the probability of correctly responding “old” to targets minus the probability of incorrectly responding “old” to novel foils (p (“old”|target)−p (“old”|foil)). This target recognition metric relies more on familiarity-based memory, typically thought to not require hippocampal (or at least DG) mediated processing^21, 22^.

### Depression scale (BDI-2)

The Beck Depression Inventory (BDI) -2, one of the most commonly used self-reported measure of depression was adopted to assess depressive mood of participants^23^. We assessed depression because depressive mood was previously shown to be associated with discrimination performance^24, 25^.

### Statistical analysis

Before conducting the study, we checked the sample size needed using a priori power analysis with the statistical software G-Power^26^. An optimal total sample size of N = 60, with a small effect size of η^2^ = 0.02 and a power of 0.8 and alpha = 0.05 was calculated.

For group analysis, we divided participants into two groups based on a median split of $\dot{{\rm{V}}}$O_2peak_. Grouping was conducted separately for each sex because $\dot{{\rm{V}}}$O_2peak_ is significantly higher in males. Participants with scores above the median were classified as the higher fitness group (High-Fit), and participants with scores below the median were classified as the lower fitness group (Low-Fit). We compared demographic variables, TPA, $\dot{{\rm{V}}}$O_2peak_ and mnemonic discrimination task performance between High-Fit and Low-Fit groups using unpaired t-tests. Then, we ran a repeated measures two-way ANOVA on 1−p (“old”|Type) with group (High-Fit, Low-Fit) × trial type (Target, High-Sim, Mid-Sim, Low-Sim, Foil) followed by Bonferroni’s post hoc test.

Next, we investigated the association of aerobic fitness and physical activity with mnemonic discrimination task performance across all participants. First, in order to determine covariates, Pearson correlation analysis was conducted to assess bivariate relationships of potential confounding variables (sex, age, BMI, BDI-2) to $\dot{{\rm{V}}}$O_2peak_, TPA, and mnemonic discrimination task performance (target recognition, and DS for high, middle, and low similarity lures). When there were meaningful correlations (r > 0.20^27^), these confounding variables were controlled as covariates for the next partial correlation analysis between $\dot{{\rm{V}}}$O_2peak_, TPA, and mnemonic discrimination task performance.

Then, we conducted mediation analyses for examining the mediation effect of $\dot{{\rm{V}}}$O_2peak_ (mediator variable) on the relationship between TPA (independent variable) and memory performance (dependent variable) using a multiple regression approach^28^ and the nonparametric bootstrapping procedure^29^. Any confounding variable exhibiting a meaningful correlation in the bivariate correlation analysis was controlled as a covariate. According to the moderation model by Baron and Kenny (1986), analysis should be conducted in four steps in order to examine the mediation effect. In the current study, the following analyses were conducted. We examined (1) whether TPA (independent variable) was associated with memory performance (dependent variable), (2) whether TPA (independent variable) was associated with $\dot{{\rm{V}}}$O_2peak_ (mediator variable), (3) whether $\dot{{\rm{V}}}$O_2peak_ (mediator variable) was associated with memory performance (dependent variable), and (4) whether the prospective mediation effect was significant when the relationship between TPA and memory performance became significantly weaker (partial mediation) or insignificant (full mediation) after the inclusion of $\dot{{\rm{V}}}$O_2peak_.

To test the significance of the mediation effect, we used the bootstrapping method recommended for relatively small sample sizes because bootstrapping is a nonparametric test that does not require the assumption of normality. In the bootstrapping method, thousands of samples are taken from a given data set and the indirect effects in each resample are estimated. These estimations are used to directly test mediation. Indirect effects can be estimated by subtracting the direct effects of the independent variable on the dependent variable after controlling for the role of the proposed mediators from the total effect of the independent variable on the dependent variable without controlling for the proposed mediators. Therefore, statistically significant mediation can be defined if zero is not included within 95% bias-corrected and accelerated confidence intervals for indirect effects. In our analyses, we used 5000 bootstrap resamples of the data with replacement.

Statistical significance was set a priori at p < 0.05. Statistical analyses were performed using SPSS Statistical Package version 19 (SPSS, Inc., USA). For mediation analysis, we used PROCESS macro for SPSS^29^.

## Results

### Group analysis (High-Fit vs. Low-Fit)

**Figure 2:**
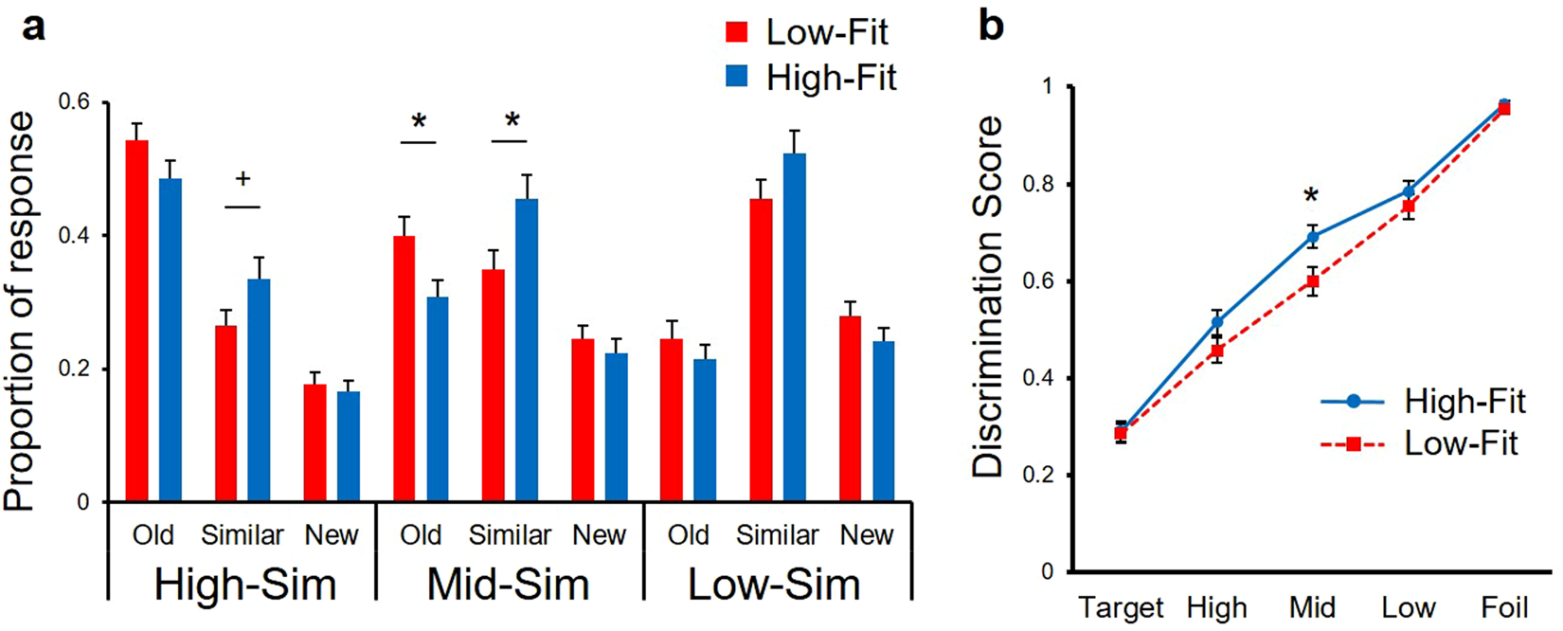
(**a**) Response proportions for each category of high-, middle- and low-similarity lures in High-Fit and Low-Fit groups. Note that response types (old, similar, new) are not independent categories. Values are mean ± SE, *p < 0.05, ^+^p < 0.1. (**b**) Mnemonic discrimination performance differences between High-Fit and Low-Fit as a function of interference levels. Discrimination score = 1 − p(“old”|Type). Values are mean ± SE, *p < 0.05.

First, we tested for group differences in mnemonic discrimination task performance. The median $\dot{{\rm{V}}}$O_2peak_ of males and females was 46.6 and 36.2, respectively. One male subject with a median $\dot{{\rm{V}}}$O_2peak_ was excluded, hence each High-Fit and Low-Fit group consisted of 37 subjects (15 females). (Note: $\dot{{\rm{V}}}$O_2peak_ range for male: Low-Fit 30.7–46.1, High-Fit 46.6–57.8; female: Low-Fit 27.3–35.6, High-Fit 36.9–45.1). Comparison and statistical testing of group differences between High-Fit and Low-Fit groups in demographic variables, physical activity, aerobic fitness and mnemonic discrimination task performance are reported in Table 1. $\dot{{\rm{V}}}$O_2peak_ and TPA were significantly higher in the High-Fit group (t(72) > 6.85, p < 0.01; t(72) > 3.54, p < 0.01) and BMI was significantly lower in the High-Fit group (t(72) = 2.08, p < 0.05). Group differences in response proportions for each similarity of lures are shown in Fig. 2a. In middle-similarity lures, the occurrence of lures identified as “similar” (correct rejections) was significantly higher in the High-Fit group (t(72) = 2.41, p = 0.02), while the occurrence of lures identified as “old” (false alarms) was significantly lower in the High-Fit group (t(72) = 2.35, p = 0.02). There is a clear trade-off between correct rejections and false alarms, that is, the High-Fit group discriminated more lures as “similar”.

Mnemonic discrimination performance differences between the High-Fit and Low-Fit groups as a function of interference levels are presented in Fig. 2b. A repeated measures two-way ANOVA for group and trial type exhibited a significant main effect of trial type (F(4, 288) = 597.67, p < 0.001), no main effect of group (F(1, 72) = 2.41, p = 0.12), and a significant interaction (F(4, 288) = 2.87, p = 0.03). Bonferroni-corrected post hoc comparisons revealed that the High-Fit group significantly outperformed the Low-Fit group on DS for Mid-Sim (F(1, 288) = 5.82, p = 0.02). Meanwhile, the High-Fit group did not differ from the Low-Fit group on DS for Target (F(1, 288) = 0.02, p = 0.88), High-Sim (F(1, 288) = 2.40, p = 0.12), Low-Sim (F(1, 288) = 0.81, p = 0.37) or Foil trials (F(1, 288) = 0.84, p = 0.36). Together, these results suggest that the better discrimination performance by the High-Fit group relative to the Low-Fit group is specific to trials with moderate mnemonic interference.

### Correlation analysis

**Figure 3:**
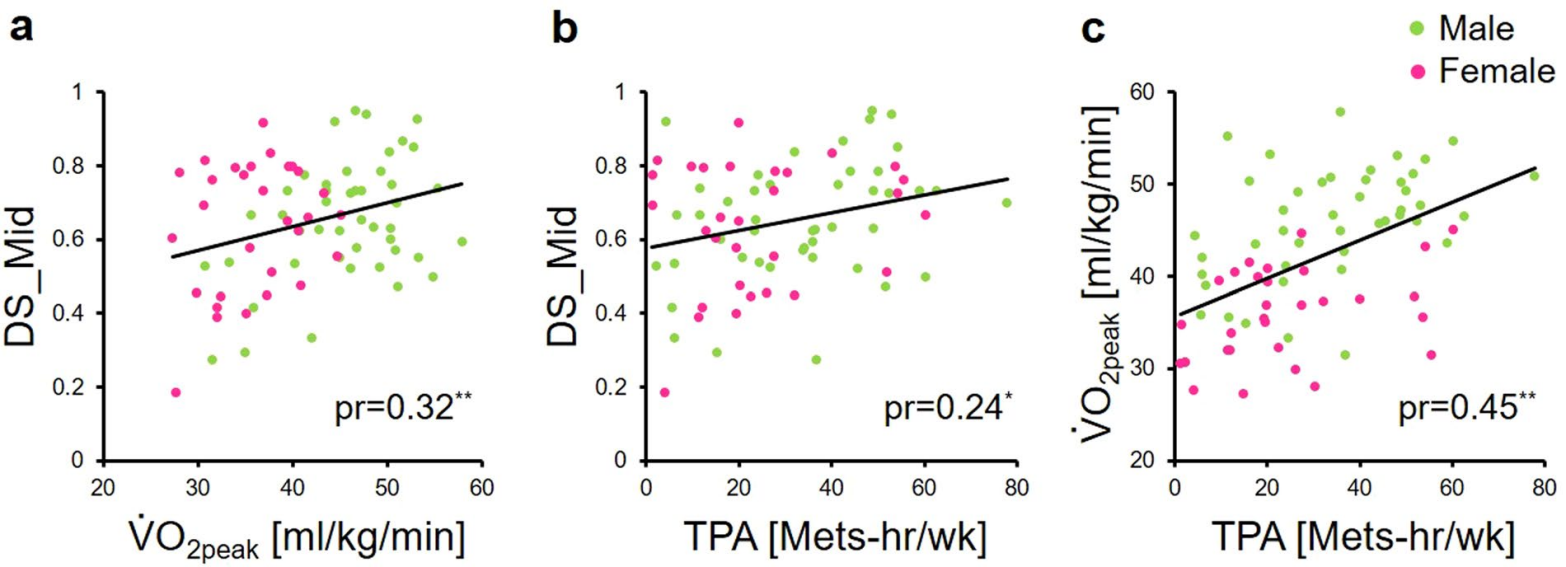
(**a**) Relationship between aerobic fitness ($\dot{{\rm{V}}}$O_2peak_) and discrimination score for middle-similarity bins (DS_Mid). (**b**) Relationship between physical activity and discrimination score for middle-similarity bins. (**c**) Relationship between physical activity and aerobic fitness ($\dot{{\rm{V}}}$O_2peak_). Discrimination score = 1 − p(“old”|Lure), pr = partial correlation coefficient after controlling for sex. *p < 0.05.

The results of bivariate and partial correlation analysis are shown in Table 2. Sex was significantly related to $\dot{{\rm{V}}}$O_2peak_ and TPA. Age, BMI and BDI-2 were not correlated with $\dot{{\rm{V}}}$O_2peak_, TPA or mnemonic discrimination task performance (all r < 0.2). Therefore, sex was controlled as a covariate for $\dot{{\rm{V}}}$O_2peak_ and TPA in the next partial correlation and regression analysis. Partial correlation analysis revealed positive correlations of $\dot{{\rm{V}}}$O_2peak_ and TPA to DS for middle-similarity bins (DS_Mid) after controlling for sex (Fig. 3b,c). DS for the other similarity bins (High-Sim and Low-Sim) and target recognition scores were not associated with $\dot{{\rm{V}}}$O_2peak_ and TPA. In addition, TPA was positively correlated with $\dot{{\rm{V}}}$O_2peak_ (Fig. 3a).

**Table 2:** Summary of correlation and partial correlation analysis for demographic data, physical activity, fitness, and memory performance.

	Fitness (V·O_2peak_)	Physical Activity (TPA)	Target Recognition	Discrimination Score		
				High	Mid	Low
Sex	−0.629**	−0.251*	−0.070	0.055	−0.054	−0.144
Age	−0.136	−0.169	0.077	0.033	−0.043	−0.013
BMI	0.013	0.057	0.172	0.020	0.100	0.037
BDI-2	−0.087	−0.090	0.085	−0.088	−0.185	−0.035
V·O_2peak_^#^	—	0.445**	−0.140	0.186	0.323**	0.198
TPA^#^	—	—	−0.215	0.119	0.240*	0.007

Note: BDI = Beck Depression Inventory; $\dot{{\rm{V}}}$O_2peak_ = Peak Oxygen uptake; TPA = Total Physical Activity. Values are Pearson’s product-moment correlation coefficient. ^#^Partial correlation coefficient controlled for sex as a covariate, *p < 0.05, **p < 0.01.

### Mediation analysis

**Figure 4:**
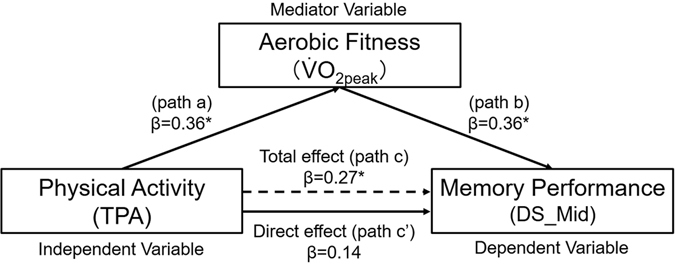
Mediation model. Aerobic fitness ($\dot{{\rm{V}}}$O_2peak_) as a mediator of the effect of physical activity on discrimination score for middle-similarity lures (DS_Mid). Path c: total effect of TPA on DS_Mid; path a: effect of TPA on $\dot{{\rm{V}}}$O_2peak_; path b: effect of mediator on DS_Mid; path c′: direct effect of TPA on DS_Mid through a mediator. Sex was entered as a covariate for all paths. β indicates standardized regression coefficient. *p < 0.05, **p < 0.01.

Figure 4 and Table 3 show results of mediation analyses with sex as a covariate. TPA was significantly associated with DS_Mid (Fig. 4 and Table 3 path c) and $\dot{{\rm{V}}}$O_2peak_ (Fig. 4 and Table 3 path a). When $\dot{{\rm{V}}}$O_2peak_ was added as the predictor, it was significantly associated with DS_Mid (Fig. 4 and Table 3 path b) and the significant association between TPA and DS_Mid diminished and became not significant (Fig. 4 and Table 3 path c’). This mediation effect was further examined using non-parametric bootstrapping procedures. The 95% bootstrap confidence intervals of the indirect effect (path a*b) did not contain zero (standardized indirect effect: 0.13, 95% CI: 0.023 to 0.278), indicating significant mediation. These results further confirm the mediation effect of $\dot{{\rm{V}}}$O_2peak_ on the relationship of TPA to DS_Mid. Thus, this mediation model suggests that the relationship between physical activity and discrimination performance is mediated by aerobic fitness levels.

**Table 3:** Results of mediation analysis.

	R^2^	ΔR^2^	B	SE B	*β*	t value	ΔF
**IV to MV (path a)**							
Model	0.519**						18.56
TPA			0.151	0.035**	0.364	4.31	
Sex			−8.223	1.293**	−0.537	−6.36	
**Total effect of IV on DV (path c)**							
Model	0.071						5.26
TPA			0.002	0.001*	0.269	2.29	
Sex			0.005	0.040	0.013	0.11	
**Direct effect of MV on DV (path b) & IV on DV (path c’)**							
Model	0.132*	0.061*					5.00
TPA			0.001	0.001	0.139	2.24	
$\dot{{\rm{V}}}$O_2peak_			0.008	0.004*	0.356	1.09	
Sex			0.069	0.048	0.205	1.43	

Note: R^2^ = coefficient of determination; B = unstandardized regression coefficient; SE = standard error. *p < 0.05, **p < 0.01.

## Discussion

The goal of the present study was to clarify whether aerobic fitness, a physiological marker reflecting habitual physical activity, is associated with discrimination performance, especially when mnemonic interference levels are moderate, in healthy young adults. In group comparisons, the High-Fit group outperformed the Low-Fit group selectively in DS_Mid. This relationship was confirmed by correlational analyses across all participants, in which aerobic fitness ($\dot{{\rm{V}}}$O_2peak_) and physical activity levels measured with IPAQ were positively correlated with DS_Mid, but not with target recognition and DS for other similarity conditions. In addition, mediation analysis clearly showed that $\dot{{\rm{V}}}$O_2peak_ and TPA predicted DS_Mid and that the effects of TPA were mediated by $\dot{{\rm{V}}}$O_2peak_. These results support the hypothesis that aerobic fitness, as a mediator of physical activity effects, is associated with discrimination performance when interference is moderate, which is consistent with predictions from computational models and suggests that aerobic fitness is associated with memory flexibility.

**Figure 5:**
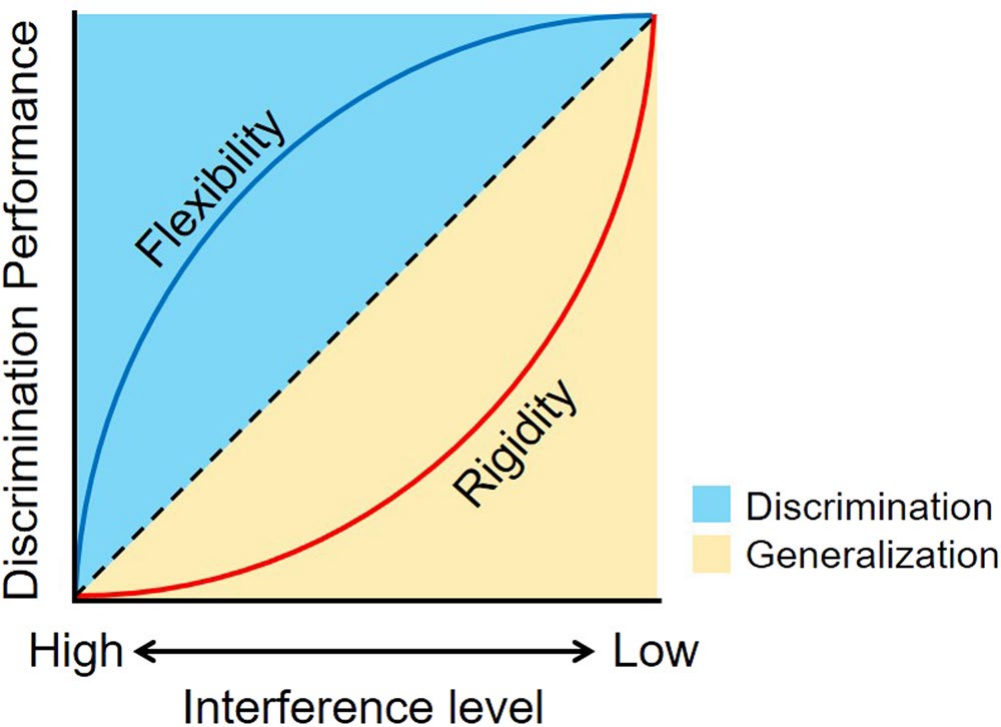
Conceptual model of memory rigidity and flexibility. Memory rigidity is observed with loss of DG function and loss of pattern separation as seen in aging and in loss of neurogenesis and other models of pathology. Conversely, memory flexibility is expected with enhancement of DG function and enhanced pattern separation.

Examining performance as a function of interference shows a linear relationship between interference and performance in the Low-Fit group, and curvilinear relationship in the High-Fit group approximated by the natural logarithmic function (Fig. 5: memory flexibility). The opposite result has been obtained with age-related mnemonic discrimination deficits, where older adults underperform young adults in mnemonic discrimination score for middle-similarity lures, namely exhibit a function approximated by an exponential curve^15, 17^ (Fig. 5: memory rigidity; note that the term “rigidity” was first used by Gallagher and colleagues^30^). A recent high-resolution fMRI study revealed that the exponential relationship of mnemonic interference and discrimination performance is linked to reduced DG/CA3 ability to generate different representations for moderate interference stimuli in older adults^18^. This evidence strongly suggests that memory rigidity is attributed to the deficit of DG-mediated pattern separation. Taken together, the association observed in the present study can be attributed to exercise-enhanced modulation of DG-mediated pattern separation.

Subsequent correlation analysis supported the above results that aerobic fitness and physical activity were associated with DS_Mid. Previous cross-sectional studies have demonstrated the positive association of aerobic fitness levels^3, 4, 31–33^ and physical activity levels^33^ with hippocampal memory function using a relational memory paradigm. Only one previous study indicated that discrimination performance changes after exercise training were positively correlated with improved aerobic fitness in a small sample of young adults^24^. However, this study did not examine discrimination performance as a function of interference level, and thus could not directly examine input/output transformations that are necessary to test hypotheses about pattern separation. Our results confirm and extend prior work, while testing a hypothesis that better discrimination performance in individuals with higher aerobic fitness manifests as memory flexibility, which has never been demonstrated.

Moreover, in a correlation analysis, we found a positive relationship between physical activity and aerobic fitness, and confirmed the validity of aerobic fitness as a physiological landmark of physical activity^14^. Mediation analysis further indicated that aerobic fitness mediated the relationship between physical activity and discrimination performance for moderate mnemonic interference levels. These results are in line with the cardiovascular fitness hypothesis, which states that cardiovascular fitness is a physiological mediator that predicts various aspects of cognitive functioning and that enhancements in aerobic fitness are necessary for the cognitive benefits of physical activity^34, 35^.

As expected, target recognition in the mnemonic discrimination task was not associated with aerobic fitness and physical activity. Previous studies have shown that the target recognition metric of the mnemonic discrimination task relies more on extrahippocampal cortices^15, 21^. Supporting this, amnesic patients with hippocampal damage demonstrate intact target recognition performance, but an impairment in lure discrimination performance using a mnemonic discrimination task^22^. Taken together with these previous findings, the current data suggest that the impact of physical activity and aerobic fitness on episodic memory is linked specifically to DG computations, and have little or no relationship with extrahippocampal cortices. These results are consistent with previous studies showing that aerobic fitness is associated with hippocampal-related relational memory, but not with hippocampal non-related item memory performance in healthy young adults and children^3, 32^.

Regarding the neurobiological mechanisms underlying the association of higher physical activity levels with better hippocampal memory function through the mediation of higher aerobic fitness levels, physical-exercise-enhanced neural plasticity of the DG may provide some insight. Previous animal studies, including our own works, have revealed a beneficial effect of exercise, especially on neurogenesis in the DG^5–9^. Moreover, an exercise-induced increase in the number of newborn neurons was correlated with enhanced fine spatial, suggesting that neurogenesis may play a role in better discrimination performance^36^. To explore this point, developing novel approaches to measure neurogenesis in the human brain *in vivo* will be necessary. Some progress has been made in this arena using MR spectroscopy techniques^37^ but the method remains in its infancy and is not without its critics^38–42^. Measuring related biomarkers such as, DG/CA3 functional activity^18^ or perforant path integrity^18, 43^, as a modifiable neurobiological basis for the effect of exercise, can also be informative. In addition to its impact on hippocampal plasticity and neurogenesis, exercise clearly has more diverse effects on the brain and can also positively impact other cognitive capacities such as executive function, mediated by the prefrontal cortex (PFC). Several studies, including our own, have shown that aerobic fitness and physical activity are associated with dorsolateral-PFC (DLPFC) function^44–46^. Interestingly, recent work has also suggested that cortical regions including the DLPFC may contribute to pattern separation^47^. While this is certainly a possibility that cannot be excluded, the selectivity of the exercise findings in this study to the middle-similarity lures suggest that the effect is at least principally driven by DG computations. To explore this in detail, however, BOLD signal changes in the DG as well as PFC regions, including the DLPFC, should be examined using high-resolution fMRI.

We should note that the mediation model, while consistent with a causal effect of exercise on DG structure and function, is not sufficient to infer such causality. Since the current study has a cross-sectional design, we are unable to draw a causal conclusion. However, many animal studies have revealed that exercise has beneficial effects on the structure and function of the DG^36^. In addition, our recent work in humans has revealed that acute physical exercise of moderate intensity improves discrimination performance when interference level is moderate, which is consistent with a theoretical hypothesis of memory flexibility^48^. Collectively, the extant data are consistent with a causal effect of exercise on brain structure and function. In other words, we would suggest that aerobic fitness improved with physical activity enhances DG-related memory function. Testing this hypothesis more directly using neurobiological approaches including high-resolution imaging is a logical next step. Moreover, further studies should clarify whether the relationships observed in the non-athlete, healthy young adults in this study can also be observed in other populations, such as children and older adults, and in psychiatric disorders, such as depression and mild cognitive impairment (MCI). It would also be interesting to consider discrimination performance in endurance athletes with superior aerobic fitness.

In conclusion, the present study reveals that aerobic fitness levels, as a mediator of physical activity effects, are selectively associated with enhanced mnemonic discrimination performance for moderate interference levels in young adults. While several studies have found a positive association between physical activity and aerobic fitness for hippocampal memory function, the current study points to involvement of improved DG function in this association. Although future research is required to confirm the causal relationship between aerobic exercise and DG-mediated pattern separation and to explore the underlying mechanism of this association, the present study sheds light on how physical activity and related improvements of aerobic fitness impact on hippocampal memory.
